# Corner Simulation of CMOS Analog Integrated Circuit Taking into Account Radiation Influence

**DOI:** 10.3390/mi17030300

**Published:** 2026-02-27

**Authors:** Sergei Ryzhov, Vadim Kuznetsov, Vladimir Andreev

**Affiliations:** 1 JSC “Experimental Design Bureau of Microelectronics (OKB MEL)”, 75-2, Grabtsevskoye Shosse, 248003 Kaluga, Russia; sergey.righov@gmail.com; 2 Electronic Engineering Department, Bauman Moscow State Technical University, 5, 2nd Baumanskaya Str., 105005 Moscow, Russia

**Keywords:** electronic design automation (EDA), microelectronics, RADFET, radiation effects, parameter extraction, compact model, circuit simulation

## Abstract

This paper proposes a corner analysis approach for CMOS circuits taking into the account radiation effects. The presented simulation approach is implemented using the open-source design automation (EDA) software QUCS-S 25.2.0 and Ngspice 45. It was developed a radiation-sensitive field-effect transistor (RADFET) SPICE macromodel representing threshold voltage shift versus radiation dose. The extraction procedure for this model is based on statistical measurements of pMOS transistors and process corner models (Slow, Typical, Fast) and involves percentile analysis. The article proposes an original design of the RADFET-based radiation sensor with RADFET device and CMOS readout circuit placed on the same die, which allows us to simplify the dosimeter schematic. The sensor output parameter dependency on process parameters, supply voltage, and temperature was investigated using the proposed simulation approach.

## 1. Introduction

Taking into account the influence of manufacturing process variation and fluctuations in supply voltage and temperature is essential for analog integrated circuit design [1,2,3]. The classic approach to analyzing these influences is corner analysis or PVT analysis, derived from the words Process, Voltage, and Temperature [4]. Specialized mostly proprietary electronic design automation (EDA) tools such as Cadence Spectre [5] or SymSpice [6,7] are used to evaluate circuit sensitivity to these variations. A set of SPICE models taking account process parameter variation [8] must be provided for this analysis type as part of process design kit (PDK). The usage of open-source EDA tools and PDKs like IHP OpenPDK [9] or Google Skywater PDK is becoming a trend of the recent years. It has been shown that open solutions may serve as a replacement for proprietary software [10,11].

The design of radiation-hardened ICs [12] is an important task for areas such as radiation therapy medical equipment, aerospace and satellite equipment design. RADFET sensors readout circuits [13,14,15] are another case where the radiation effects must be taken into the account. Most of the present works on this subject are dedicated to digital circuit design [16,17,18] or consider the analog design of digital cells [19]. The design of radiation-hardened analog ICs is not fully covered in the available publications and presents an actual task. For radiation-hardened or radiation sensing analog circuits, ionizing radiation can also be considered as a corner parameter while performing PVT analysis. This is because radiation exposure alters the parameters of both active and passive circuit components, and these changes superimpose on the variations caused by PVT [4]. Circuit simulation software based on SPICE does not provide a unified way to introduce a radiation influence in the standard SPICE devices library. The common approach is to develop a semiconductor device model taking into account radiation effects. This task may be solved by Verilog-A compact model development [20,21,22] or using a more simple approach involving standard SPICE model modification [23,24]. All the models proposed in [20,21,22,24] are developed for a single “typical” or “nominal” device and do not incorporate the process variation of the technology. For instance, work [24] presents averaged characteristics, with results shown as single, smooth curves without indicating sample-to-sample variations. In works [20,21], the SPICE model parameters are extracted from measurements of specially fabricated test transistors with fixed dimensions, neglecting variations between different devices. A similar approach is demonstrated in [24]. The results show good agreement with experimental data. However, parameter variation between individual devices is not considered. Therefore, these models do not assess the potential spread in characteristics. Thus, existing radiation-aware SPICE models describe the average behavior of a device but provide no information on the process-induced spread of its characteristics.

Therefore, while these models are valuable for predicting nominal post-irradiation behavior, they are fundamentally incapable of predicting the statistical spread of circuit performance. This represents a significant gap in the design flow for radiation-hardened circuits, as it leaves designers blind to the yield impact of process variation under radiation.

The novelty of the presented work is the following:

1. It introduces a process corner analysis approach specifically for the RADFET device. This allows taking into account the manufacturing process variation and fluctuation influence, supply voltage and temperature variance.
2. It integrates this radiation-aware corner analysis into a holistic PVT simulation flow using the open-source tools QUCS-S and Ngspice, at a level comparable to commercial EDA tools.
3. It was proposed a radiation sensor design when the both RADFET sensor and readout circuit are placed on the same die. The benefit of the proposed design is that all transistors may be formed in the single chip as matched pairs and no external current source is required for sensor readout. This allows us to simplify the analog part of the dosimeter schematic.

The main purpose of this research is to investigate the feasibility of this approach and provide a practical workflow for evaluating the combined impact of process variation, voltage, temperature, and ionizing radiation on circuit performance.

The rest of the paper is organized as follows. Section 2 provides a detailed description of the used approach to represent the RADFET model and considers a procedure for corner parameter extraction of the MOSFET model for the technology node used. Section 3 considers corner simulation of the current mirror circuit used for readout of the RADFET sensor and gives a result comparison for proprietary and open-source circuit simulation tools. Finally, Section 4 gives an outcome of the paper and the summary of the achieved results.

## 2. RADFET Model Design

### 2.1. Simulation Software and Technology Node Used

QUCS-S is an open-source cross-platform circuit simulator [11,25,26] with a powerful EDA with intuitive GUI and support for modern simulation engines, namely Ngspice [27], Xyce [28] and QucsatorRF. The key feature of QUCS-S is an advanced performance for RF circuit design [10,29] and out-of-the-box tools for microelectronic schematic implementation and compact model debugging, including the Verilog-A synthesizer [22].

QUCS-S allows fast switching of the SPICE-compatible simulation backend, and the Ngspice simulation engine was selected for this research. Ngspice is an open-source simulator operating in command line (CLI) mode. It is actively integrated into modern open PDKs, such as IHP Open PDK [30] and Skywater 130A PDK [31]. Ngspice is used for simulating Hot Carrier Degradation, Single-Event Upset (SEU), Single-Event Effects (SEEs), and aging [31].

In this article, the corner models were developed using data from a Parametric Control Monitor (PCM) [32] fabricated on a single semiconductor wafer in full compliance with the CD4000 integrated circuit manufacturing technology, which is also used for the RADFET [33,34]. This technology corresponds to a long-channel CMOS process with minimum channel lengths of about 5 μm. The selection of the micron technology node is required by the compatibility of the readout circuit with the RADFET sensor technology. The submicron technology has a very limited application for RADFET sensor design because a relatively thick gate dielectric is required for sensor operation. IV curve measurements from 16 pMOS transistor samples with fixed dimensions of *L* = 5 μm and *W* = 100 μm were used. Based on this data, the parameters for a SPICE model Level 1 [35] were extracted for each transistor. These parameters are the following: threshold voltage VTO (V), transconductance KP ($A/V^{2}$), bulk threshold parameter GAMMA ($\sqrt{V}$), and channel length modulation LAMBDA (1/V). The parameter extraction method was similar to that described in [36,37].

The corner models were selected using the following procedure. A sorted series was constructed for each of the SPICE model parameters VTO, KP, and LAMBDA. And the distribution percentiles were calculated based on these series. The 10th and 90th percentiles were used for the “Slow” (PMOS_SS) and “Fast” (PMOS_FF) corner definitions, respectively. The typical model (PMOS_TT) was selected as the one whose parameters were closest to the median values of the distributions. This procedure corresponds to a multivariate statistical corner selection approach, where representative devices are chosen from the joint parameter distribution rather than constructed by independent parameter scaling, as commonly applied in statistical circuit analysis. Such multivariate selection preserves physically consistent parameter combinations and is widely used in statistical circuit analysis [2,32,38]. In this context, the term “worst-case” refers only to the most extreme parameter combinations among the extracted corner models and does not imply a statistically defined worst-case of the entire technological process and are not intended to represent statistically defined PDK corners or to be used for yield analysis.

The RADFET corner model was constructed using parameter deviations derived from pMOS transistors because the RADFET sensor structure is fully compatible with the basic technology node and differs only by device geometry. Therefore, no additional corner parameter measurement is required for the RADFET device. In micron technologies the influence of geometry on the parameter spread is significantly lower than in deep submicron technologies [39]. Therefore, the obtained variation coefficients can be used as an approximate estimate of the process spread for transistors of the same technology.

### 2.2. PMOS Corner Models

The parameters of the PMOS_SS corner are lower than those of the typical PMOS_TT corner by approximately 0.83% for the KP parameter and 9.02% for the Lambda parameter, and higher by 0.40% for the VTO parameter. Conversely, the parameters of the PMOS_FF corner are lower by 12.78% for the KP parameter and 0.01% for the VTO parameter, and higher by 4.25% for the Lambda parameter. These deviation values will be used further to construct the RADFET transistor macromodel.

**Figure 1:**
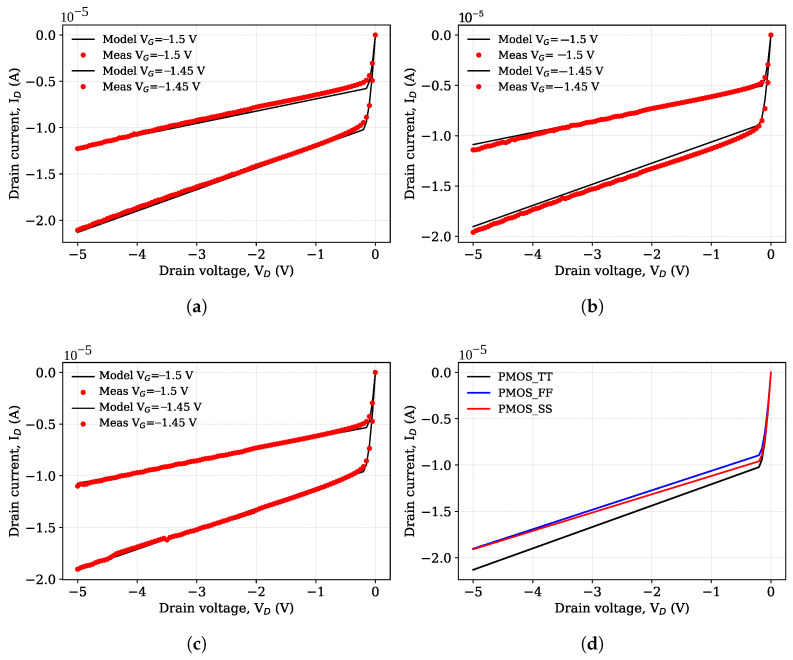
Output I-V characteristics of pMOS transistors for different process corners: (**a**) Typical corner (PMOS_TT). (**b**) Fast corner (PMOS_FF). (**c**) Slow corner (PMOS_SS). (**d**) PMOS corner comparison at V_*G*_ = −1.5 V.

Figure 1 shows a comparison of the output I-V characteristics of the pMOS transistors for the different corner models.

The typical corner corresponds to a median representative point in parameter space and therefore is not required to exhibit electrical characteristics lying between PMOS_SS and PMOS_FF models.

**Figure 2:**
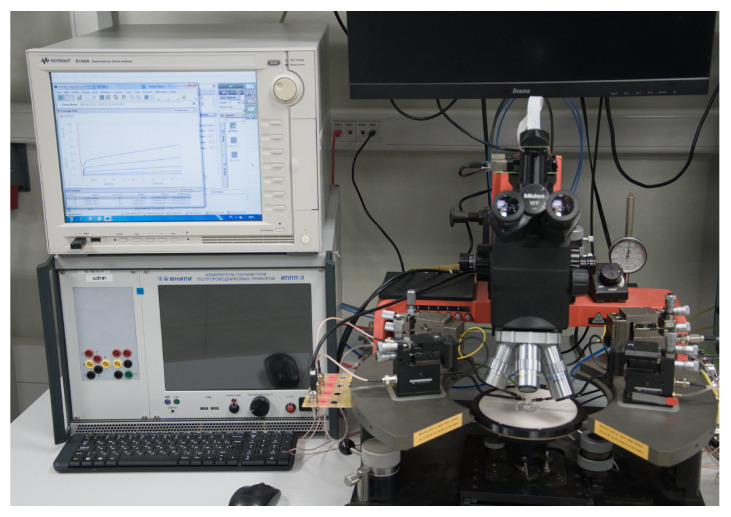
Semiconductor devices I-V curve measurement setup.

The I-V curves of the PMOS transistors were measured using the Keysight B1500A semiconductor device analyzer at a nominal temperature of 27 °C. Figure 2 shows the photo of the measurement setup. It consists of a B1500A curve tracer itself and the wafer probe station connected to it and it allows us to measure the I-V curves of the samples on the wafer.

### 2.3. RADFET SPICE Macromodel Design

The RADFET is a MOS transistor acting as an ionizing radiation dose sensor. The operation principle of the RADFET devices is based on the threshold voltage shift dependency on the accumulated radiation dose because of charge trapping in the gate dielectric [33]. Typically the PMOS transistors are serving as RADFETs.

The RADFET does not belong to the standard SPICE primitive device set and requires the design of a custom model. There exist several approaches to resolve this task. The research [22] proposes a compact model representing effects such as nonlinear dependency of threshold voltage shift on accumulated dose, saturation, fading, and device operation under gate bias up to the Fowler–Nordheim injection mode [40,41]. Another more simple approach is the modification of the existing SPICE MOSFET model by passing the threshold voltage shift on the accumulated dose as the model parameter. For example, the research [20] uses the EKV v2.6 model [37,42] as the basic model and adds empirical dependencies for the model parameters VTO, GAMMA, KP, and E0 on the accumulated dose. This approach will work for every SPICE model representing the RADFET for the basic technology node. In the present article, we have used a Level 1 SPICE model as the basic RADFET sensor model and added the dependency of threshold voltage of the accumulated dose, because the full simulation of the RADFET sensors, including the effects mentioned above, was not required. A similar approach was presented by a group of researchers from Nis University in their article [24].

The macromodel was constructed using measurement data on the threshold voltage shift of a p-channel MOSFET with *W*/*L* = 700 μm/6 μm versus the radiation dose taken from [22,33]. The initial threshold voltage was −1.2 V. A threshold voltage dependency on the accumulated dose was approximated by linear function as proposed by [24]. This approximation could be described by the following equation:

(1) $$\Delta V_{th}=V_{th0}+D\cdot S$$

where $V_{th0}$ is the initial threshold voltage, *D* represents the accumulated dose expressed in rads, *S* is the sensor’s sensitivity to radiation expressed in V/rad.

The parameter value $S=6.16\times 10^{-5}$ V/rad was determined using the least squares method, resulting in an root mean square error (RMSE) value of $2.63\times 10^{-11}$.

**Figure 3:**
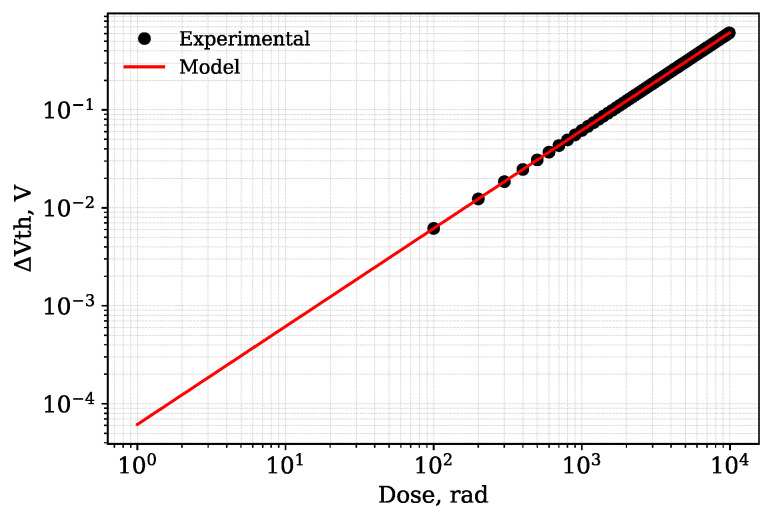
Threshold voltage shift versus accumulated dose for the RADFET.

Figure 3 shows the log-log plot of the threshold voltage shift versus the accumulated dose. The experimental data are represented by markers, while the approximating curve is shown as a solid line. The selected RADFET sensor operates in the region where this dependency may be considered as linear.

## 3. Corner Simulation Taking into Account Radiation Effect

### 3.1. Current Mirror Circuit for Biasing RADFET

There exist different approaches to design the readout circuit for the RADFET sensor. Usually the RADFET sensor should be connected to current source IC (for example LM334) after the exposure to measure the threshold voltage shift. The dosimeter design presented in [43] uses this circuit. We propose another approach for readout circuit design based on using a current mirror. The usage of the current mirror has an advantage that it uses the same technology node for the RADFET and the readout circuit. This makes it possible to design a chip set containing the RADFET and its readout circuit.

**Figure 4:**
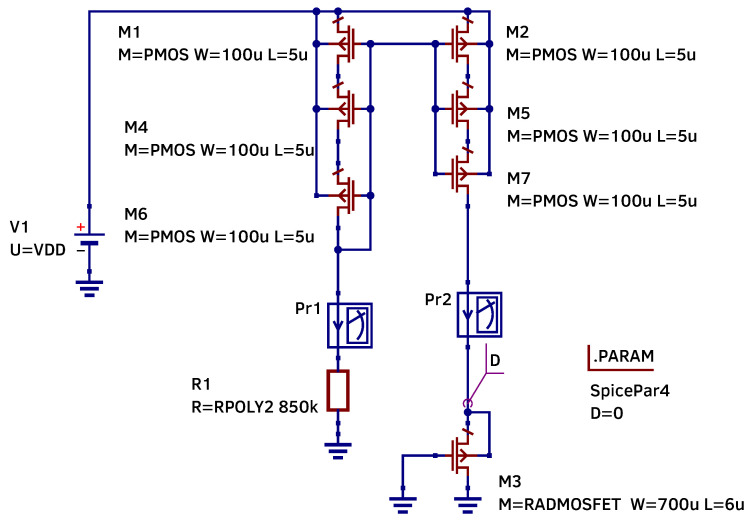
Current mirror with the RADFET transistor in QUCS-S.

The current mirror schematic which is used for our research is shown in Figure 4. It consists of two PMOS stacks: M1, M4, M6 from the reference branch and M2, M5, M7 from the output branch. The polysilicon resistor R1 defining the current is connected to one branch of the mirror. The RADFET sensor M3 is connected to another branch. The threshold voltage of the RADFET sensor is measured at the mirror output (node labeled D–drain). The .PARAM block defines the parameter variable *D* representing the accumulated dose. This variable is passed to M3 instance parameters.

To improve current matching and reduce the impact of channel-length–dependent threshold voltage variation, each PMOS transistor in the current mirror is implemented as a series connection of identical shorter transistors with their gates tied together, following the approach described in [3].

The simulation of the RADFET transistor’s threshold voltage shift under ionizing radiation was implemented using the nested loop parameter sweep of the *D* variable and supply voltage DC sweep. The accumulated dose *D* is swept in a range from 0 to 10 krad. The supply voltage is swept in a range from 9 to 11 V which corresponds to a 10% supply variation.

The SPICE model library contains three sets of polysilicon resistor models (minR, maxR, typ) and three sets of PMOS transistor models (PMOS_TT, PMOS_SS, PMOS_FF). The polysilicon resistor models were obtained using measurements from 32 samples. The process variation for the RADFET was implemented using multipliers k1, v1, and l1, which were derived from the current mirror pMOS transistors. The content of the library is shown in the Listings 1 and 2.

**Listing 1.** SPICE model library for polysilicon resistors.
* Typical corner

.LIB res_typ

.MODEL RPOLY2 R(RSH=1400, TC1=-0.0014, TC2=3.6E-6 TNOM=27)

.ENDL

* min RSH corner

.LIB minR

.MODEL RPOLY2 R(RSH=1200, TC1=-0.0014, TC2=3.6E-6 TNOM=27)

.ENDL

* max RSH corner

.LIB maxR

.MODEL RPOLY2 R(RSH=1600, TC1=-0.0014, TC2=3.6E-6 TNOM=27)

.ENDL


**Listing 2.** SPICE model library for pMOS transistors and RADFET.
* pMOSFET library

.LIB PMOS_TT

.param k1=1 v1=1 l1=1

.param S=6.16e-05 VT=1.2

.MODEL PMOS PMOS (Level=1 VTO=-1.2925 KP=2.302e-05 LAMBDA=0.2364 GAMMA=0.290

+ TNOM=27)

.MODEL RADMOSFET PMOS (Vt0=-(VT*v1+D*S) Kp=(5m*k1) Gamma=3.97u

+ Phi=0.6 Lambda=(1.87m*l1) Tox=100n TNOM=27)

.ENDL


.LIB PMOS_SS

.param k1=0.9917283727656981

.param v1=1.0040197249360854

.param l1=0.90984315841993

.param S=6.16e-05 VT=1.2

.MODEL PMOS PMOS (Level=1 VTO=-1.2977 KP=2.283e-05 LAMBDA=0.2151 GAMMA=0.290

+ TNOM=27)

.MODEL RADMOSFET PMOS (Vt0=-(VT*v1+D*S) Kp=(5m*k1) Gamma=3.97u

+ Phi=0.6 Lambda=(1.87m*l1) Tox=100n TNOM=27)

.ENDL


.LIB PMOS_FF

.param k1=0.8721575100916387

.param v1=0.9999170424701207

.param l1=1.0425197000956137

.param S=6.16e-05

.param VT=1.2

.MODEL PMOS PMOS (Level=1 VTO=-1.2924 KP=2.007e-05 LAMBDA=0.2465 GAMMA=0.290

+ TNOM=27)

.MODEL RADMOSFET PMOS (Vt0=-(VT*v1+D*S) Kp=(5m*k1) Gamma=3.97u

+ Phi=0.6 Lambda=(1.87m*l1) Tox=100n TNOM=27)

.ENDL


The following procedure was used to evaluate the impact of process variation, temperature, and supply voltage fluctuations on the performance of the circuit under investigation.

The analysis was conducted at the following extreme corner combinations:

- Temperature: −60 °C and 125 °C;
- Supply voltage: 9 V and 11 V;
- Resistor models: minR and maxR;
- Transistor models: PMOS_FF and PMOS_SS.

For each combination, radiation exposure simulation was performed with a dose ranging from 0 to 10 krad. The results were compared with the typical corner set (27 °C, 10 V, PMOS_TT, res_typ).

### 3.2. Simulation Results

The performed simulations show the impact of various corners on the output voltage stability of the investigated circuit under radiation. The most significant parameter is temperature with a maximum deviation of 10.59% at 125 °C.

Process variations and supply voltage fluctuations demonstrated a smaller impact: no more than 0.03% for supply voltage fluctuations within a range of ±0.5 V and 0.4% for process variations.

Table 1 provides a summary of the output voltage deviations obtained from the corner analysis.

**Figure 5:**
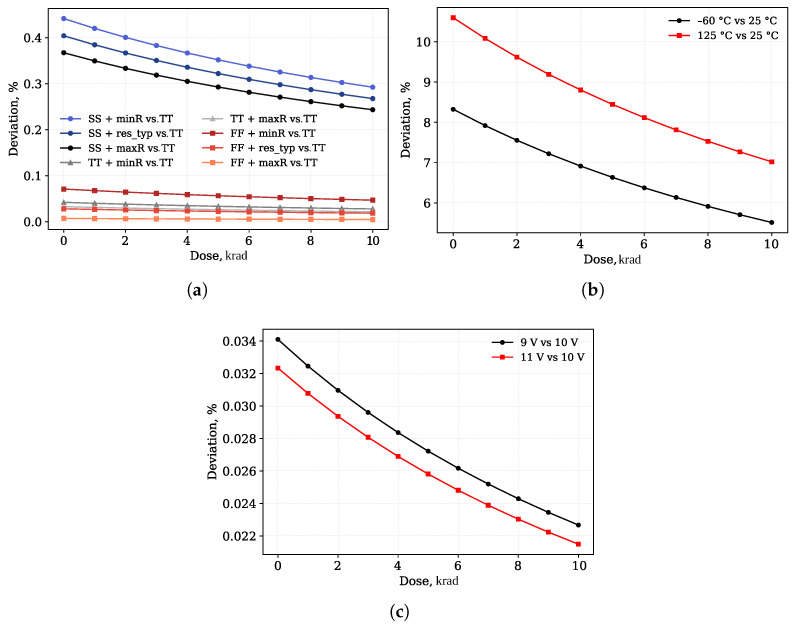
Relative deviation of output voltage from nominal corner versus absorbed radiation dose for different conditions: (**a**) process corners (relative to TT), (**b**) temperature (relative to 25 °C), (**c**) supply voltage (relative to 10.0 V).

The relative deviations of the output voltage from the nominal corner depending on radiation dose for different corners are shown in Figure 5.

**Figure 6:**
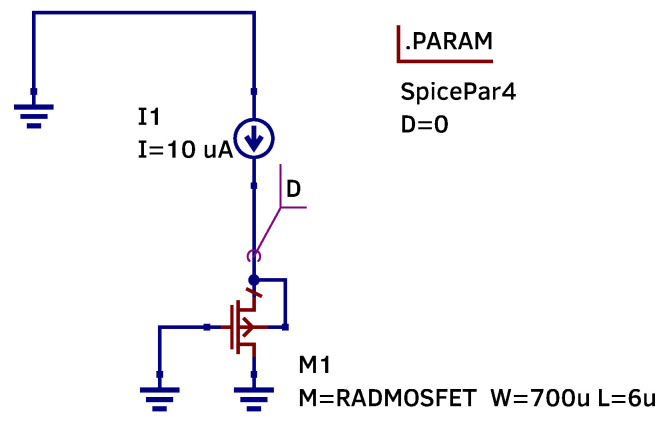
Simplified circuit using an ideal current source to bias the RADFET.

To evaluate the impact of the corner parameters on the RADFET, a circuit with an ideal current source was simulated. The ideal current source eliminates dependence on supply voltage and any variations associated with the formation of the reference current. The simulated circuit is shown in Figure 6.

**Figure 7:**
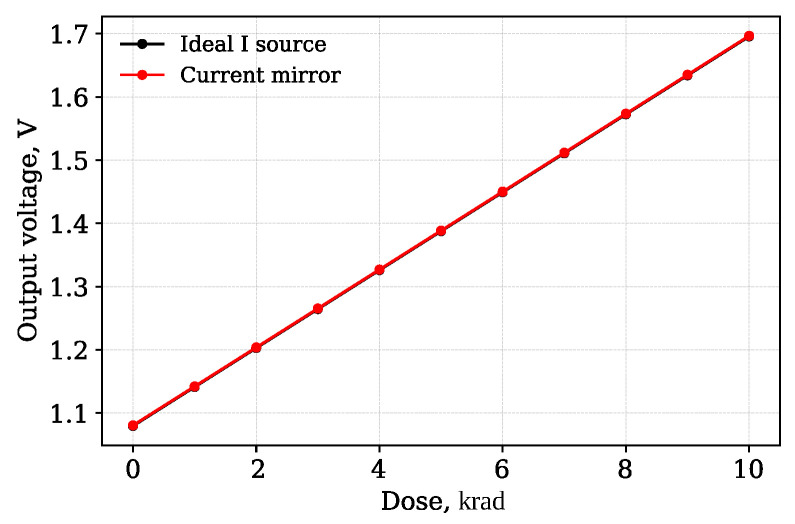
Comparison of RADFET simulation results with an ideal current source biasing and a current mirror biasing at the worst-case temperature corner.

For comparison of the simulation results, Figure 7 shows the simulation results of the RADFET at the worst temperature corner (125 °C) under two conditions: with an ideal current source and with a current mirror.

As shown in Figure 7, the differences between the two circuit designs are minor. The maximum deviation is 0.09% indicating that the biasing method has negligible influence.

The observed temperature sensitivity can be explained by the physical properties of MOS transistors. Even when the RADFET is biased by an ideal current source (Figure 6), temperature remains the dominant factor affecting output voltage stability. Specifically, temperature has an effect on two key parameters: carrier mobility (μ) decreases, while threshold voltage ($|V_{th}|$) also decreases with temperature. Although $|V_{th}|$ reduction slightly increases drain current, this effect is dominated by the dominant mobility degradation. SPICE simulations confirm that the mobility degradation dominates this competition, resulting in an overall decrease in output voltage with temperature.

The operating point was simulated and internal parameters were obtained for a RADFET using the circuit diagram shown in the Figure 6.

However, NGSPICE does not directly calculate parameters such as carrier mobility (μ) and threshold voltage ($|V_{th}|$). Therefore, two related quantities were extracted instead: the $V_{on}$ parameter (turn-on voltage) and the normalized transconductance. SPICE simulators assume $V_{on}\approx V_{th}$ for the most of practical cases. The normalized transconductance is defined as

(2) $$g_{m_{norm}}=\frac{g_{m}}{\left(W/L\right)}$$

where $g_{m}$ is the small-signal transconductance and (W/L) is the transistor’s aspect ratio.

For long-channel devices operating in strong inversion, $g_{m}$ can be approximated as

(3) $$g_{m_{norm}}\approx \mu \cdot C_{ox}\cdot \left(V_{gs}-V_{th}\right)$$

From this relation, it follows that $g_{m_{norm}}$ is directly proportional to carrier mobility and is ideal for demonstrating temperature dependence.

**Figure 8:**
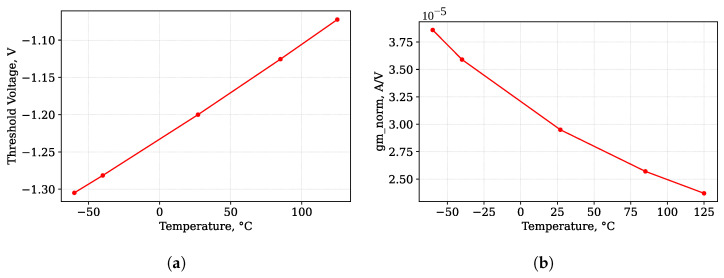
Results of the operating point analysis: (**a**) Threshold voltage vs. temperature. (**b**) Normalized transconductance vs. temperature.

The simulation results shown in Figure 8, illustrate the dependence of the threshold voltage and normalized transconductance on temperature.

The effect of temperature in the SPICE Level 1 models used is taken into account using built-in analytical temperature relationships [39]. Only the nominal reference temperature (TNOM) is specified in the parameters, while the operating temperature is set globally, and the simulator automatically recalculates the transistor parameters according to the model’s internal temperature dependencies.

### 3.3. Deterministic Estimation of Current Mirror Mismatch

In addition to the process variations in corner models, local transistor mismatch is a significant source of uncertainty for current mirrors. Mismatch refers to the local spread of parameters between nominally identical transistors and arises from random microscopic fluctuations in the process [44]. For current mirrors, mismatch results in differences in the electrical characteristics of transistors, which causes the output current to deviate from the nominal value even with identical device parameters.

The model described in [44] requires knowledge of the process mismatch coefficients $A_{VT}$ and $A_{\beta }$, which, as shown, are strictly process-dependent quantities and must be extracted experimentally. This model is a generally accepted empirical description of local mismatch in MOS transistors and is widely used as an analytical basis for specifying random parameter variations in Monte Carlo simulations of analog integrated circuits [2,45]. Furthermore, the relative current spread in current mirrors is determined not only by the mismatch coefficients but also by the transistor’s operating mode: according to the results of [44], the contribution of the mismatch threshold voltage to the relative current spread decreases with increasing overdrive voltage $V_{ov}$.

In this article, a deterministic worst-case mismatch estimate is used to evaluate the impact of mismatch on a current mirror circuit. The simulation uses the inclusion shown in Figure 7, where the current deviation is accounted for through the equivalent change in the ideal source current. Conservative upper bounds for the mismatch coefficients from [44] were used in the calculations. These estimates provide experimental data for a wide range of CMOS technologies, including technologies with an oxide thickness of 100 nm, which corresponds to the used CD4000-compatible technology.

The effect of mismatch on current is considered for two boundary values:

(4) $$I=I_{nom}\left(1\pm 3\sigma_{I}/I\right)$$

where $I_{nom}$ is the nominal value of current and $\sigma_{I}/I$ is the standard deviation of current due to mismatch.

This approach provides a rapid estimate of the circuit sensitivity to mismatch at the initial stage of analysis and is widely used during early design phases when statistically characterized process models are unavailable. It does not replace full Monte Carlo simulation with characterized SPICE models containing mismatch parameters.

The relative current variation $\sigma_{I}/I$ was estimated using the classical Pelgrom mismatch model (5) from [44].

(5) $$\sigma_{I}/I=\frac{1}{\sqrt{WL}}\sqrt{\frac{4A_{VT}^{2}}{V_{ov}^{2}}+A_{\beta }^{2}}$$

For this purpose, the minimum possible overdrive voltage within the operating PVT conditions of the circuit was considered. This condition corresponds to the PMOS_TT corner combined with minimum load resistance, a supply voltage of 9 V, and a temperature of −125 °C.

The obtained value of $\sigma_{I}/I$ = 0.06. The dominant contribution is exerted by $\sigma_{V}t$ = 2.5 mV.

**Figure 9:**
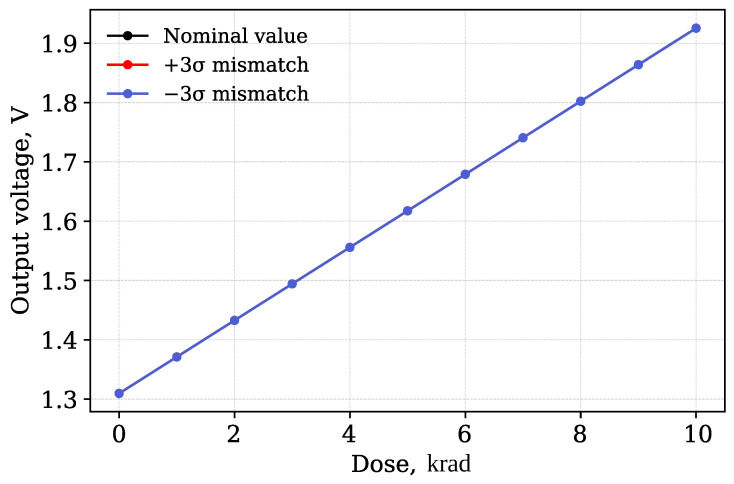
Output voltage dependency on accumulated dose for nominal and ±3σ mismatch conditions.

It should also be noted that radiation exposure can further enhance the mismatch of transistor parameters. As shown in [46], the total ionizing dose leads to an increase in the parameter spread due to the combined effect of technological variations and differences in the electrical modes of the transistors during irradiation. Thus, mismatch in a radiation environment is a function not only of the technological spread but also of the cumulative dose. In this study, the radiation-induced contribution to mismatch is not separately modeled and is not considered as an area for further research. In this study, for each considered radiation dose, a separate calculation of the standard deviation of the parameters was performed, which made it possible to take into account the dependence of mismatch on the cumulative dose. The Figure 9 shows shows the dependence of the output voltage on accumulated radiation dose for the upper and lower bounds of the mismatch variation.

The maximum relative output voltage deviation due to transistor parameter mismatch was determined as the largest ratio of the difference between the ±3σ levels and the nominal value to the nominal level over the entire range of doses studied. It was found that the relative mismatch does not exceed 8.4 × 10^−5^ V.

### 3.4. Simulation of RADFET Sensor and Readout Circuit Placed on the Same Die

**Figure 10:**
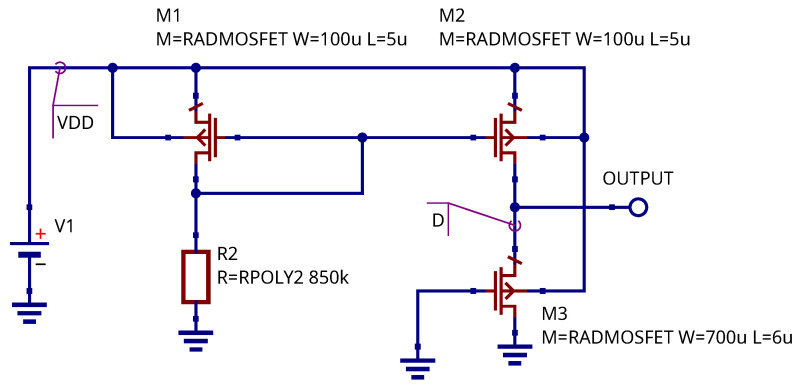
RADFET sensor combined with readout circuits; all transistors are exposed to radiation.

In Section 3.1 we have considered a case when the RADFET sensor and readout circuit were formed separately and connected only at the readout phase. We propose an approach when the readout circuit and sensor are placed in the same chip. The usage of current mirror as the readout circuit makes this design possible. The schematic representing the proposed solution is presented in the Figure 10. The key differences from the schematic shown in the Figure 4 is that all three MOSFETs are exposed to radiation. The PMOS transistors M1 and M2 serves as a current source, and PMOS transistor M3 serves as the radiation sensor. The polysilicon resistor R1 is also exposed to radiation, but the degradation of its parameters may be neglected. It was shown in the research in [47,48] that the degradation of polysilicon films starts from doses higher than $10^{6}$ rads. This value is out of the typical RADFET sensor operating range.

**Figure 11:**
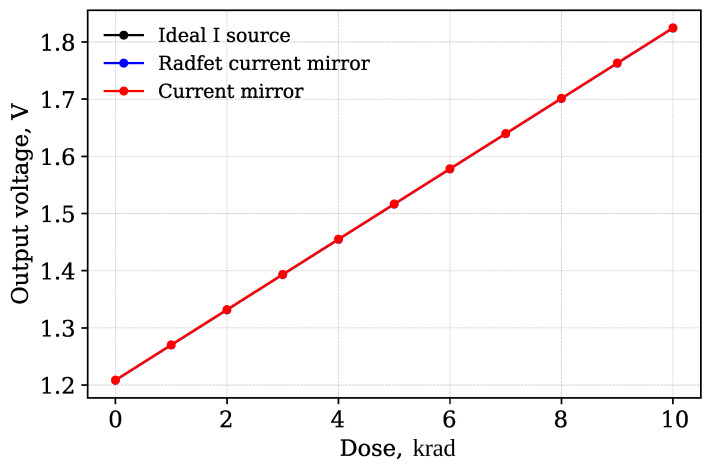
RADFET The dependency of sensor output voltage in absorbed dose.

The sensor output is the drain of M3 transistor (node label D). The voltage at this node has a linear dependency on the accumulated dose. Figure 11 shows the simulated dependency of the sensing circuit output voltage (node D) on the accumulated radiation node. This dependency is compared to the dependency obtained for the ideal current source readout circuit and non-irradiated current mirror. The comparison shows no visible difference if all four transistors placed on the same die and irradiated at the same time. The observed difference lies within 1% tolerance.

**Figure 12:**
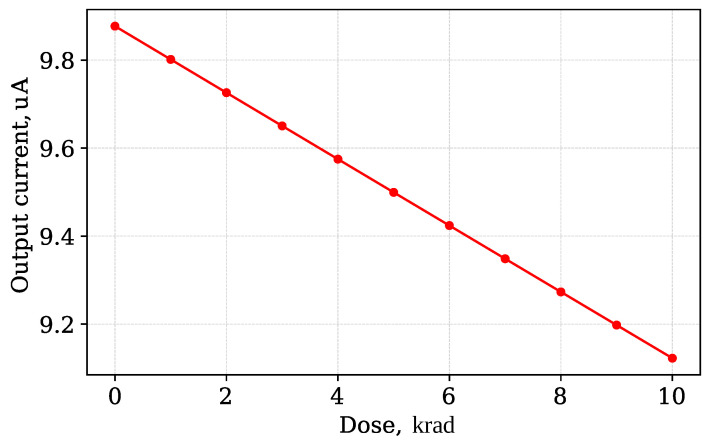
M3 MOSFET drain current dependency on accumulated dose.

The drain current of M3 MOSFET (output current) has a weak dependency on the radiation dose (Figure 12), because the threshold voltage shift of the M1 and M2 transistors (current mirror) is compensated. It could be seen that the M3 drain current changes within 10% for the dose changed within the sensing circuit operating range. This current may be set near the ZTC point of the M3 transistor. The simulation shows that the difference of the measured threshold voltage shift using ideal current source and proposed sensor schematic is within 0.1% over the operating range from 0 to 10 krad.

Summarizing all the above, we can conclude that the proposed circuitry allows us to form the RADFET sensor and readout current source in a single chip. This monolithic IC will not require an external current source for readout and its output could be connected directly to the ADC input of a digital dosimeter.

The proposed RADFET sensor and readout circuit were simulated under the same PVT corner combinations as described earlier. Table 2 summarizes the difference between the two implementations of the readout circuit.

The simulation results demonstrate that the circuit in which only the RADFET sensor is exposed to radiation and the circuit in which all transistors are exposed exhibit almost identical sensitivity to PVT variations. Temperature variation remains the dominant corner (approximately 10%), whereas the influence of process and supply voltage variations stays below 0.4% and 0.04%, respectively. These results support the feasibility of integrating both the RADFET sensor and its readout circuit on the same die. Also it should be noted that the RADFET used inside the readout circuit had geometry different from the device used during SPICE model extraction. However, for the used micron-scale technology, this approximation does not introduce significant inaccuracy.

### 3.5. Simulation Result Comparison with Proprietary EDA Tools

**Figure 13:**
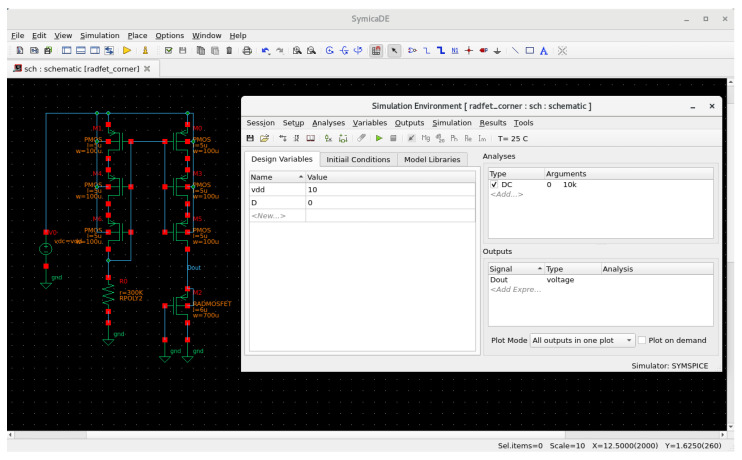
Screenshot of configuration in the SymicaDE environment.

**Figure 14:**
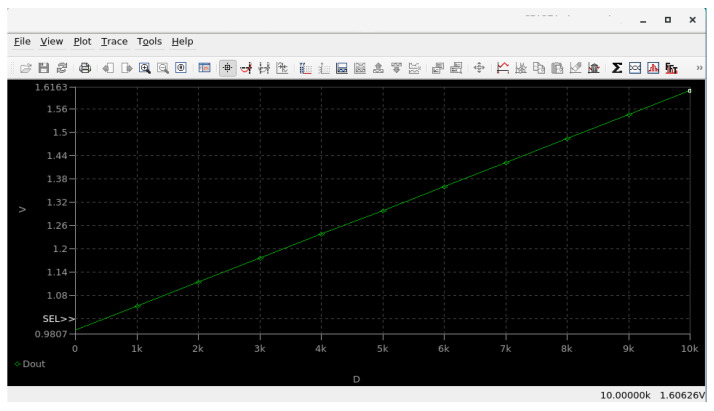
Screenshot of simulation results in the SymicaDE environment.

To validate the accuracy of the performed simulations using open-source EDA, a comparative analysis was conducted with the commercial EDA SymicaDE v5.5. This software package uses Spectre-compatible netlist syntax and a primitive devices library and could be used for analog IC design for micron and submicron technology nodes from the DC to RF domains [7]. The same circuit netlist and corner models were used in both simulation environments. However, a minor modification was required for the threshold voltage parameter due to differences in SPICE model implementation between the simulators. In the netlist for SymicaDE, the initial threshold voltage was defined as .param VT = -1.2. Figure 13 and Figure 14 show screenshots of the simulation session in SymicaDE, confirming the equivalent setup and parameter configuration.

**Figure 15:**
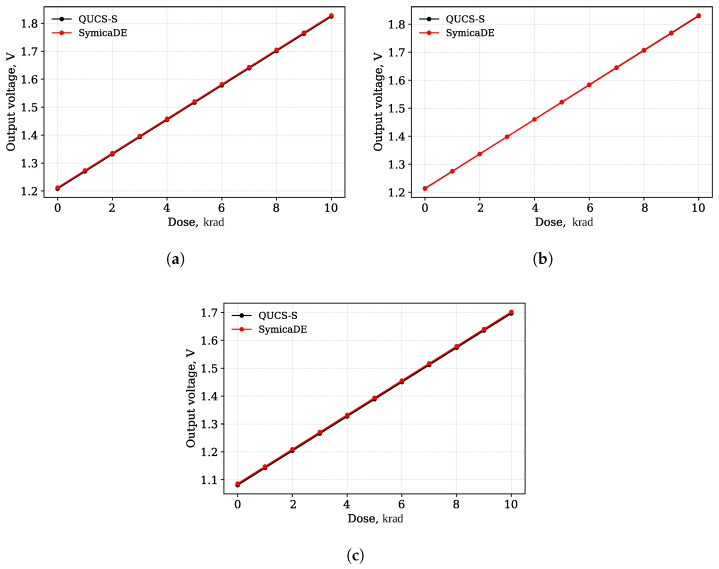
Comparison of simulation results between QUCS-S/Ngspice and SymicaDE: (**a**) Output voltage vs. radiation dose for the voltage worst corner (9 V). (**b**) Output voltage vs. radiation dose for the process worst corner (PMOS_SS/minR). (**c**) Output voltage vs. radiation dose for the temperature worst corner (125 °C).

Figure 15 shows the simulation results of the RADFET at the worst corners.

The maximum observed deviation in the output voltage between the two tools was less than 1% across all worst-case corners with the maximum deviation for temperature being 0.49%.

## 4. Conclusions

This article proposes a complete corner analysis approach for a radiation-hardened analog circuit, implemented entirely with open-source EDA. The results show good agreement with simulations performed in commercial EDA SymicaDE. Simulation results confirm the effectiveness of the QUCS-S/ngspice combination for comprehensive corner analysis, including radiation effects. The percentile-based extraction and scaling of SPICE model parameters (VTO, KP, LAMBDA) represents a practical workflow for integrating radiation-aware corners into existing open-source circuit simulators.

Our analysis reveals that the dominant corner affecting circuit is temperature. The influence of process variation and supply voltage was significantly lower. This result underscores the critical role of a holistic approach that concurrently considers temperature, radiation, and process corners.

The original radiation sensor circuitry has been proposed and simulated using the presented approach. The key feature of the proposed sensor design is that the both the RADFET sensor and current mirror readout circuit are placed on the same die. This solution allows us to simplify the dosimeter circuit.

The SPICE level 1 MOSFET model (Shichman–Hodges) provides a simple extraction procedure and good representation of MOSFET device behavior for micron technology nodes. However, several limitations related to the usage of the level 1 model should be noted. The temperature dependencies in this model are defined in a completely analytical manner, without additional SPICE parameters such as TCV, BEX, KT1 for the EKV model if we used it as the core model. These simplifications are acceptable for the technology node used in the paper but would require refinement for predictive use and extension to other types of RADFET devices in production-grade design. For more complex models like EKV-RAD or BSIMSOI RAD, radiation impact statistics collection for multiple RADFET devices, special current source design development, and taking into account the influence of radiation may be required to improve the simulation accuracy.

## Figures and Tables

**Table 1:** Summary of corner analysis.

Parameter	Max. Deviation, %	Mean Deviation, %	Worst Case
Corner model	0.44	0.14	PMOS_SS/minR
Temperature	10.59	7.67	125 °C
Voltage supply	0.03	0.02	9V

**Table 2:** Difference between simulation results of the two current mirror implementations.

Parameter	Max. Deviation, %	Mean Deviation, %	Std. Deviation, %
Corner model	−0.0035	−0.0021	−0.0002
Temperature	0.0144	0.01321	−0.0026
Voltage supply	−0.0028	−0.0023	−0.0006

## Data Availability

Source modeling data uploaded via Zenodo: https://doi.org/10.5281/zenodo.17952925 (accessed on 16 December 2025).
